# MiRNAs and Cancer: Key Link in Diagnosis and Therapy

**DOI:** 10.3390/genes12081289

**Published:** 2021-08-23

**Authors:** Yu Shi, Zihao Liu, Qun Lin, Qing Luo, Yinghuan Cen, Juanmei Li, Xiaolin Fang, Chang Gong

**Affiliations:** 1 Breast Tumor Center, Sun Yat-Sen Memorial Hospital, Sun Yat-Sen University, Guangzhou 510000, China; shiy77@mail2.sysu.edu.cn (Y.S.); liuzh75@mail2.sysu.edu.cn (Z.L.); linq27@mail2.sysu.edu.cn (Q.L.); luoq63@mail2.sysu.edu.cn (Q.L.); cenyh@mail2.sysu.edu.cn (Y.C.); lijm95@mail.sysu.edu.cn (J.L.); fangxlin3@mail.sysu.edu.cn (X.F.); 2 Guangdong Provincial Key Laboratory of Malignant Tumor Epigenetics and Gene Regulation, Sun Yat-Sen Memorial Hospital, Sun Yat-Sen University, Guangzhou 510120, China

**Keywords:** microRNA, cancer, diagnosis, therapy

## Abstract

Since the discovery of the first microRNA (miRNA), the exploration of miRNA biology has come to a new era in recent decades. Monumental studies have proven that miRNAs can be dysregulated in different types of cancers and the roles of miRNAs turn out to function to either tumor promoters or tumor suppressors. The interplay between miRNAs and the development of cancers has grabbed attention of miRNAs as novel tools and targets for therapeutic attempts. Moreover, the development of miRNA delivery system accelerates miRNA preclinical implications. In this review, we depict recent advances of miRNAs in cancer and discuss the potential diagnostic or therapeutic approaches of miRNAs.

## 1. Introduction

Hippocrates naming the abnormal mass of tissue in stomach and uterus as “cancer” revealed the long-lasting exploration of human malignant tumors [1]. The International Agency for Research on Cancer (IARC) has published the Latest Global Cancer Data, which indicates that in 2020, nearly 19,300,000 people were diagnosed with cancer worldwide. Breast cancer has the highest incidence among 36 types of cancer, while the mortality of lung cancer remains the highest. Due to its forementioned characteristics, cancer has remained the top leading cause of death worldwide for nearly a decade [2]. Still, efforts have been made to protect humans from cancer. The development of diagnostic and therapeutic strategies has come to a new era. The current clinical diagnostic and therapeutic approaches are diverse, and new genomic diagnostic tools as well as therapeutic systems are under development [3,4].

Since the discovery of the first microRNAs (miRNAs) on Caenorhabditis elegans in 1993, the understanding of miRNA biology has grown deeper. The action of mechanisms of miRNAs, how they participate in the regulation of genes in prokaryotes and eukaryotes, have been revealed [5,6]. miRNAs are capable of binding to the 3′UTR of corresponding mRNA, which ultimately leads to the post-transcriptional suppression or degradation or silencing of target genes [7]. It has been revealed that approximately 30% of human genes can be regulated by miRNAs [8]. Moreover, a single miRNA can interact with a dozen of mRNAs, which ultimately influence the expression of many genes simultaneously [9]. The complex regulation function of miRNAs is involved in multiple biological process, including cell proliferation, differentiation, invasion, migration, and apoptosis [10]. Meanwhile, these dysregulated biological processes are bound up with sets of diseases.

As a result, miRNAs relate to miscellaneous human disease including tumors [11,12,13]. The very first discovery of a cancer associated with miRNA was published in 2002 [14]. Since then, growing studies have demonstrated the abnormal expression of miRNAs in several types of tumors [15,16,17,18]. In this review, we mainly focus on the relationship between miRNAs and cancer biogenetic process as well as miRNA-based diagnostic and therapeutic strategies. 

## 2. miRNA Biology

miRNAs are small endogenous RNAs, 19–25 nucleotides in length. In 1993, the very first miRNA lin-4 was found in Caenorhabditis elegans, and it potentially suppresses the expression of lin-14, lin-28, lin-41, lin-42, and daf-12 [19,20,21]. Several decades later, nearly 3000 miRNAs have been found in the human genome. miRNAs have been proven to take part in the normal cell growth as well as the development and progression of diseases. As the fundamental element of RNA-induced silencing complex (RISC), miRNAs modulate target gene expression by the degradation of targets or inhibition of target gene translation [22,23,24]. 

Generally, miRNAs are biosynthesized by two pathways. In the canonical biosynthesis pathway, primary miRNAs (pri-miRNAs) are transcribed from the introns by RNA polymerase II (RNA Pol II). Then, the microprocessor complex, composed of Drosha enzyme and DiGeorge syndrome critical region 8 (DGCR8), interacts with pri-miRNA to cut the specific stem-loop structure to 60–70 nt in size and processes pri-miRNAs into precursor miRNAs (pre-miRNAs) [25]. The transporter protein Exportin-5 transports pre-miRNAs to the cytoplasm. However, knockout of Exportin-5 cannot completely block the transportation of pre-miRNA. This indicates there are other transporting mechanisms to translocate pre-miRNAs into cytoplasm [26,27]. At last, the Dicer enzyme, composed of TAR RNA-binding protein (TRBP) and protein activator of the interferon-induced protein kinase (PACT), can splice pre-miRNA into single-strand mature miRNA and load mature miRNA with AGO protein into RISC [25,28]. In the noncanonical pathway, miRNA biosynthesis is independent of the microprocessor. Mirtrons directly derive from introns and ignore the interplay with the microprocessor complex, while the remaining procedures remain the same [29].

**Figure 1:**
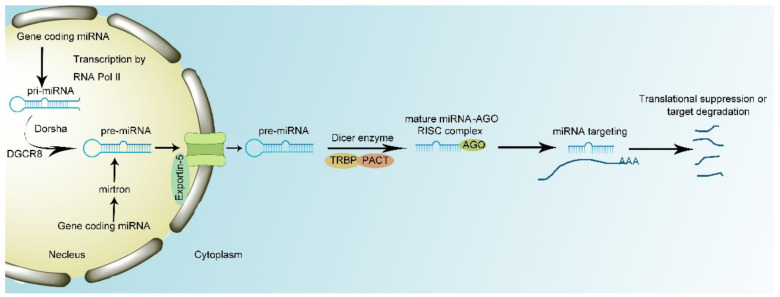
miRNA biogenesis and mechanism of action. miRNA production remains conserved across species. RNA polymerase II transcribes pri-miRNA from genome sequence. Drosha and its cofactor protein bind to primary miRNAs (pri-miRNA) leading to the excision of the loop structure to generate precursor miRNA (pre-miRNA). Then Exportin-5 transports pre-miRNA from nucleus to cytosol. Dicer complex, composed of TAR RNA-binding protein (TRBP) and protein activator of the interferon-induced protein kinase (PACT), manipulates maturation of miRNA and formation of RNA-induced silencing complex (RISC) complex. Mature RISC complex binds to target mRNA with complementary sites, resulting in the translational suppression or target degradation.

Once biosynthesized, miRNAs complementarily base pair with corresponding mRNA, a process that is initiated by conformational change of the MID and PIWI domains in AGO proteins [30]. miRNA interacts with 3′UTR of target mRNA to manipulate its expression. The seed region of miRNA which is composed by the 2nd–7th of the nucleotides of miRNA sequence conducts the interaction between miRNA and mRNA. mRNAs with an adenine opposite miRNA nucleotide 1 (t1A) ahead of seed-region-target sequences have the maximum binding capacity. Moreover, the 13th–16th nucleotides of 3′UTR of miRNA serve as supplemental pairing region to mediate additional interaction between miRNAs and mRNAs [31,32]. miRNAs can lead to either the degradation or translation repression of target mRNA at specific site. Moreover, RISC complex can recruit polyA-nuclease deadenylation complex subunit 2/3 (PAN2/3), carbon catabolite repressor protein 4 (CCR4), and NOT complex and the recruitment of these proteins result in the degradation of mRNAs conducted by 5′-3′ exonuclease 1 [33]. In addition, miRNAs interact with eukaryotic initiation factor 4 A- I/II in the translation–initiation complex to tamper the translation of target mRNA without influencing mRNA stability [34,35]. However, recent research has proven that miRNAs can act as nuclear activating miRNAs to modulate transcription. For example, when miR-24-1 is transfected into cells, the surrounding genes’ transcription is shown to be enhanced (Figure 1) [36].

Moreover, the regulatory function of miRNAs can be influenced by competing endogenous RNAs (ceRNAs), such as circular RNAs (circRNAs), pseudogene transcripts, and long non-coding RNAs (lncRNAs). Accordingly, mounting evidence has indicated that ceRNAs play a critical role in the functional regulation of miRNAs [37,38,39]. Once bound with miRNAs, ceRNAs occupy with individual miRNA and inhibit interaction between miRNAs and target mRNA, ultimately blocking miRNA function [40]. 

## 3. miRNAs Regulate the Hallmarks of Cancers

Recent studies have expanded our understanding of the interplay among tumor initiation, progression, recurrence, and the biogenetic markers of angiogenesis, therapy-resistance, invasion, as well as metastasis activation [41]. When compared with normal tissue, miRNAs are often dysregulated in tumors. The abnormally expressed miRNAs have been proven to manipulate stemness, angiogenesis, proliferation, apoptosis process, cell cycle, and the epithelial–mesenchymal transition (EMT) of tumor cells. miRNAs regulate individual target genes directly. Moreover, one miRNA can regulate several target genes, which means a single miRNA modulates a brunch of biological functions.

Cancer stem cells (CSCs) share similar characteristics with stem cell and are capable of escaping from conventional cancer therapies. Fibroblast growth factor 2 (FEF2), as one member of the FGF family, responds to injury and tissue repair in normal neural development [42]. Recent studies have proven that FEF2 modulates the differentiation and proliferation of stem cells [43,44]. The existence of glioblastomas cancer stem cells (GSCs) largely contributes to the poor clinical outcome of patients. GSCs secret a disintegrin and metalloproteinase domain-like protein decysin 1 (ADAMDEC1), which in turn accelerate the release of FGF2. FGF2 binds to FGF receptor 1 (FGFR1) and activates Zinc-finger E-box-binding homeobox 1 (ZEB1) which exerts its transcriptional modification through the downregulation of miR-203. The overexpression of miR-203 decreases the expression of ADAMDEC1 and reduces the expression of stem-cell associated transcription factor SOX2 [45,46]. miR-34a binds to serine/threonine-protein kinase D1 (PRKD1) and suppresses the expression of stem cell marker, such as CD24, CD44 and CD133 [47]. Other miRNAs like miR-122 [48], miR-185-3p [49], miR-200c [50], etc., have been reported to mediate cancer stem cell biological processes.

Epithelial–mesenchymal transition (EMT) is a process whereby epithelial-like tumor cells acquire mesenchymal features and tend to invade or migrate. During EMT, several epithelial markers are involved, such as E-cadherin, ZO-1 and mesenchymal markers such as N-cadherin, vimentin, and fibronectin. Signaling pathways including transforming growth factor (TGF), fibroblast growth factor, Wnt/β-catenin, Notch, and human epidermal growth factor receptor have been illustrated to regulate EMT [51]. Programmed Cell Death 7 (PDCD7) can be targeted by miR-134 and it modulates E-cadherin expression through binding to the promotor of E-cadherin. The upregulation of miR-134 reduces PDCD7 and attenuates PDCD7-mediated tumor suppression [52]. Moreover, miR-1246 directly targets GSK-3β/β-catenin pathway which downregulates E-cadherin expression and promotes EMT [53]. Furthermore, miRNAs such as miR-128-3p [54], miR-190 [55], miR-612 [56], and miR-33a-5p [57] have been proven to be involved in the EMT process. The EMT process not only leads to the invasion and migration of tumor cells, but also results in therapy resistance in cancer patients [58]. The overexpression of miR-410 enhances EMT and radiotherapy resistance by increasing DNA damage repair and regulates EMT through PTEN/PI3K/mTOR axis which leads to the radiotherapy resistance in non-small cell lung cancer (NSCLC) [59]. 

The cell cycle is a complex regulatory process that cells go through in order to exponentially proliferate. It precisely modulates the replication of whole genome and division of genomic replicates into new daughter-cells, which generally contains four stages: Gap1 (G1), Synthesis (S), Gap2 (G2), and Mitosis (M) [60,61,62]. In cancer cells, the continuous proceeding of G1-S-G2-M cycle leads to the ceaseless proliferation of tumors. The abnormal cell-cycle related proteins, such as pRB, p53, cyclin-dependent kinases (CDKs), cyclin-dependent kinase inhibitor (CDKI), and cyclins contribute to cancer initiation and progression. Cancer cells can manipulate cycle-regulatory proteins through miRNAs machinery to thrive and proliferate [61]. A low expression of miR-1258 was observed in colorectal cancer, while overexpression of miR-1258 restrains tumor proliferation. It has been shown that a high expression of miR-1258 arrests cell cycle at G0/G1 phase because miR-1258 binds to the 3′UTR of E2F8 which is a regulator of cyclin D1 (CCND1) and cyclin dependent kinase inhibitor 1A (p21) [63]. Gemini, as a critical regulator of DNA replication, inhibits DNA re-replication during S, G2, and early M phases. miR-571 could downregulate Gemini level independent of anaphase promoting complex (APC/C) in late S phase and downregulation of Gemini leads to DNA re-replication and genomic instability. Moreover, CDK2-dependent c-Myc directly interacts with miR-571 promoter and restrains miR-571 expression [64]. 

**Figure 2:**
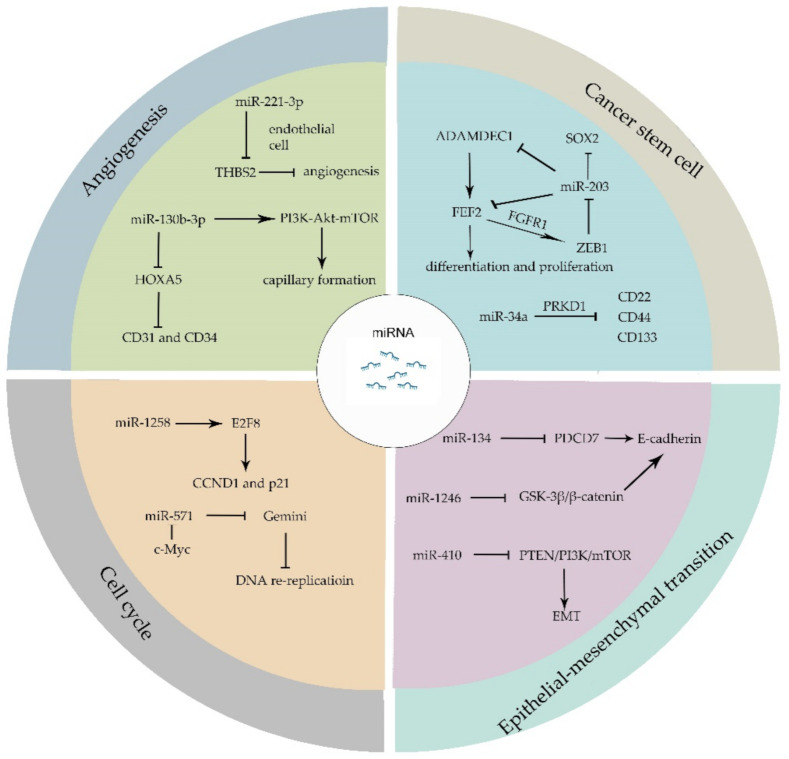
miRNAs modulate different hallmarks of cancer. miRNAs manipulate a set of biological processes that ultimately influences the proliferation and migration of cancer cells. ADAMDEC1, a disintegrin and metalloproteinase domain-like protein decysin 1; FEF2, Fibroblast growth factor 2; FGFR1, FGF receptor 1; ZEB1, Zinc-finger E-box-binding homeobox 1; PRKD1, serine/threonine-protein kinase D1; EMT, Epithelial–mesenchymal transition; PDCD7, Programmed Cell Death 7; CCND1, cyclin D1; p21, cyclin dependent kinase inhibitor 1A; THBS2, Thrombospondin-2.

Angiogenesis, as a vital process in normal cells growth and development, plays an extremely important role in tumor metastasis and survival [65,66]. Recent research works have revealed that miRNAs enroll in the regulation of angiogenesis [67,68]. The expression of miR-221-3p is related to the microvascular density and leads to tumor progression. miR-221-3p-enriched exosomes secreted by tumor cells are transferred to endothelial cells. In endothelial cells, miR-221-3p binds to the mRNA of Thrombospondin-2 (THBS2) which is regarded as angiogenesis inhibitor and inhibits THBS2 expression [69]. The upregulation of THBS2 can reverse the angiogenic effect of miR-221-3p. HOXA5, which has been proven to associate with angiogenesis biomarker CD31 and CD34, is a direct target of miR-130b-3p. The overexpression of miR-130b-3p leads to the downregulation of HOXA5 and activates PI3K-Akt-mTOR signaling pathway to provoke capillary formation (Figure 2) [70,71]. 

## 4. Therapeutic Approaches Targeting miRNA in Cancer

A variety of carcinogenesis-associated miRNAs are abnormally expressed during tumors initiation and progression [72,73]. This raises the possibility that manipulating miRNA expression might be promising for cancer treatment [9,74,75,76,77]. 

Based on the existing research, attempts to modulate the expression of miRNAs for tumors treatment are in full swing [78,79,80]. A set of exogenous miRNAs which are divided into miRNA mimics and miRNA inhibitors (antimiRs) have been developed for preclinical use. When a tumor suppressor miRNA is diminished in tumors, miRNA mimics can be introduced into tumor cells to replenish the miRNA and abolish tumor progression. miRNA mimics are often transfected to reverse specific tumor-suppressor miRNA in tumor cells. For example, in gastric and colorectal cancer cells, the overexpression of miR-451 can reduce cancer cell proliferation and increase radiotherapy sensitivity [78]. Approaches to antagonize the effects of miRNA include antisense oligonucleotides (ASOs), miRNA antagonists (antagomirs), and locked nucleic acid (LNA) -modified oligonucleotides are also well studied. ASOs are phosphorothioate nucleotides with RNase H activity and it can complement with miRNAs to suppress miRNA function, while antagomirs is a chemically modified single strand oligonucleotides that binds miRNAs to abrogate function of miRNAs [7,12,13,81]. In hepatitis virus infected mice models, an injection of antimiR-122 can significantly downregulate miR-122 level in plasma, liver, and white adipose tissue, which ultimately inhibits hepatitis C virus (HCV) replication [82,83,84,85]. Moreover, LNA-modified antimiRs have remarkable therapeutic response in mouse models [86,87].

**Figure 3:**
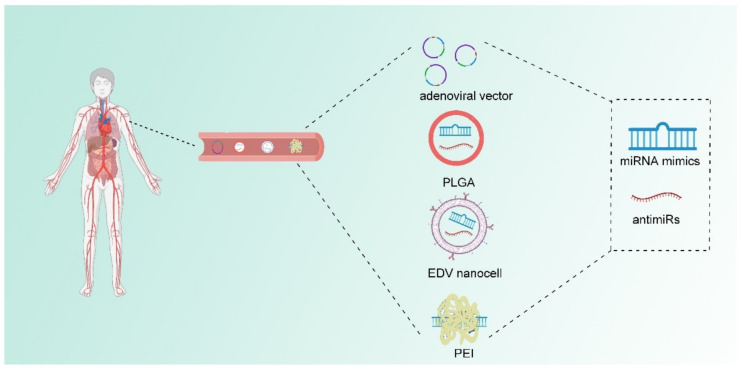
Chemical modifications and delivery system of miRNA in vivo application. The chemical modifications of miRNA mimics and antimiRs increase their stability to ensure the integrality of miRNA-based therapeutic particles, while development of delivery systems facilitates the efficiency of miRNA therapy in vivo. Some of the commonly used delivery vehicles includes adenoviral vector, Poly (lactide-co-glycolide) (PGLA), EnGeneIC Delivery Vehicle (EDV) nanocells and polyethylenimine (PEI) molecules. Safety issue as well as tumor-specific delivery systems are still tested in animal models and clinical trials.

In addition to the chemical modulation of miRNAs, delivery systems that can wrap naked nucleotides can also enhance the efficiency of exogenous miRNA because of the unstable state of naked nucleotides. Huge progress has been made to enhance in vivo delivery. Delivery systems such as adenoviral vectors, Poly (lactide-co-glycolide) (PLGA), EnGeneIC Delivery Vehicle (EDV) nanocells, and polyethylenimine (PEI) have been developed [12]. Adenoviral vectors are capable of encoding miRNA molecules, but the safety of their clinical application remains challenging [88,89]. PLGA which is currently used as biodegradable sutures show advantage for miRNA delivery in vivo. PLGA has low toxicity and can deliver RNA molecules [90,91]. EDV nanocells are 400nm particles coated with lipopolysaccharide and surface-conjugated antibodies [92,93]. A clinical trial [NCT02369198] already uses EDV nanocells to deliver miR-16 mimics in vivo [92,94]. PEI, which can form a complex with exogenous miRNA, can adhere to a negatively charged cell membrane. Studies have indicated that PEI as well as the poly (l-lysine)-modified polyethylenimine (PEI-PLL) have profound delivery efficiency in preclinical models [95,96]. Some of aforementioned miRNA delivery strategies have already implicated in clinical trials and further developments can be expected (Figure 3).

miRNA-based clinical therapy has made huge progress. Miravirsen, as one of the first miRNA-based molecules for HCV treatment clinical trial, is composed of LNA-modified miR-122 complementary sequence. Miravirsen blocks the interaction between miR-122 and HCV RNA. Moreover, Miravirsen targets pri-miR-122 as well as pre-miR-122 and it can also inhibit miR-122 biosynthesis [97]. The success of Miravirsen inspired the development of miRNA-based cancer therapies. miR-142-3p upregulates in breast cancer and leads to hyperproliferation of tumor through Wnt signaling pathway. Exosomes from bone marrow-derived mesenchymal stem cells (MSCs-Exo) were manipulated to deliver anti-miR-142-3p to tumor in vivo which efficiently suppresses cancer stem-like cells proliferation [98,99]. MRX34 is a liposomal-wrapped miR-34a mimic which is under phase I study. miR-34a interacts with programmed cell death ligand 1 (PD-L1) 3′ UTR to suppress PD-L1 expression and it is regarded as a tumor-suppressive miRNA. It has been proven that the overexpression of miR-34a can suppress a set of tumors. MRX34 might be promising for antitumor treatment. It has been validated that delivery of MRX34 in NSCLC mouse model suppresses PD-L1 level by 30%. This leads to the upregulation of CD8+T cell infiltrating rate by 38.6% and the prolonged survival of xenograft mice [100,101,102,103]. Besides, the antitumor activity of MRX34 has been tested in clinical trials which enrolled primary liver cancer, small cell lung cancer (SCLC), lymphoma, melanoma, multiple myeloma, renal cell carcinoma, and non-small cell lung cancer (NSCLC) patients [NCT01820071]. miR-16 mimics based-EDV nanocells have entered phase I trials which enrolls patients with malignant pleural mesothelioma and NSCLC [NCT02369198]. miR-155 antimiR MRG106 (Cobomarsen) have been applied in a phase I clinical trial in patients of lymphoma and leukemia [NCT02580552].

Manipulation of miRNAs can also enhance therapy sensitivity in patients. The relatively high expression of miR-621 was found to associate with better paclitaxel plus carboplatin (PTX/CBP) therapeutic effect in breast cancer patients. miR-621 suppresses FBXO11 to promote p53 activity, which consequently increases apoptosis in cancer cells. miR-621 may act as a potential therapeutic target for breast cancer treatment in the future (Table 1) [97].

However, some specific miRNAs have opposite functions in various cancers. For example, miR-106b-5p is upregulated in several cancers and proved to be oncogenic, including gastric cancer, hepatocellular carcinoma, and glioma. Vice versa, it acts as a tumor suppressor in papillary thyroid cancer, breast cancer and epithelial ovarian cancer [104,105]. As aforementioned, these specific miRNAs may likely to have adverse therapeutic effects among different cancers. The precise use of miRNA mimics or antimiRs in different cancers remains to be explored in the future.

**Table 1:** Selected miRNAs in cancers and their therapeutic manipulations.

miRNAs	Disease	Expression of miRNA	Important mRNA Target	Therapeutic Model	Reference
miR-122	HCV infection	upregulated	HCV 5′UTR	Miravirsen	[85]
miR-142-3p	breast cancer	upregulated	miR-150, APC, and P2X7R	anti-miR-142-3p	[99]
miR-34a	liver cancer, lung cancer, lymphoma, melanoma, renal cell carcinoma	downregulated	PD-L1 3′UTR	MRX34	[100]
miR-16	Mesothelioma, non-small cell lung cancer	downregulated	BCL-2, CDK1, ETS1 and JUN	miR-16 mimic	[106]
miR-155	lymphoma and leukemia	upregulated	SHIP1, WEE1, VHL, TP53INP1	AntimiR-155	[107,108,109]
miR-621	breast cancer	downregulated	FBXO11	N.A.	[97]

## 5. Diagnostic Potential of miRNA in Cancer

Since miRNAs are abnormally expressed by tumors, attempts to analyze miRNA expression profiles and detect the relative expression of miRNAs in plasma of tumor patients may predict cancer prognosis in a diagnostic manner. With the development of biotechnology, the sensitivity of detection technology has improved, and the types of detectable samples have expanded from fresh frozen tissue, paraffin tissue fixed with formalin to miRNA in body fluid, which greatly facilitates miRNA clinical applications [110,111,112]. miR-192-5p, miR-194-5p, and miR-215-5p are upregulated in Barret’s esophagus (BE). In a small cohort of 67 BE participates, researchers found that the area under the curve (AUC) to diagnose BE by combining of miR-192-5p, miR-194-5p and miR-215-5p could reach 0.96–0.97 [113]. Moreover, in a prospective study, miRNA expression in bronchial epithelium from 347 smokers were analyzed via small-RNA sequencing. The results indicate that miR-146a-5p, miR-324-5p, miR-223-3p, and miR-223-5p were downregulated in lung cancer patients. In another lung cancer cohort, the combination of miR-146a-5p and existing mRNA biomarkers profoundly improved the prediction accuracy of lung cancer [114].

Circulating miRNAs have proven to bind with argonaut protein or high-density lipoprotein (HDL), which in turn protects circulating miRNAs from degradation by extreme pH or abnormal temperature as well as RNase function. Since circulating miRNAs can be easily detected, circulating miRNAs have become potential diagnostic biomarkers in cancer [7,74]. In a cohort of epithelial ovarian cancer (EOC) patients, ovarian cystadenoma patients, and healthy women, a high level of miR-200b can predict the poor overall survival of patients [80]. Osteosarcoma which shows poor prognosis is susceptible in children and teenagers [115]. In a large training and validation cohort, 29 miRNAs have been identified abnormally expressed in osteosarcoma. Among these abnormally expressed miRNAs, miR-221 and miR-222 have been found to associate with metastasis risk of osteosarcoma and turn out to be promising prognostic markers [116]. Currently, the diagnostic biomarkers of hepatocellular carcinoma (HCC) mainly count on α-fetoprotein (AFP), *Lens culinaris* agglutinin-reactive AFP (AFP-L3) and des-γ-carboxyprothrombin (DCP). However, a recent study discovered serum miR-16 as a novel marker for HCC. miR-16 itself has a sensitivity of 72.1% and specificity of 88.8% for the detection of HCC. Moreover, when combined with AFP, AFP-L3, and DCP, the sensitivity to diagnose HCC increased to 92.4% [117]. Meanwhile, urinary miRNAs, including miR-618 and miR-650, may also be of great diagnostic value for HCC patients [118]. Plenty of studies have demonstrated that miR-155 can act as a sensitive biomarker for monitoring the change in tumor size of breast cancer, more sensitive than canonical biomarkers such as carbohydrate antigen 15-3 (CA15-3), carcinoembryonic antigen (CEA), and tissue polypeptide specific antigen (TPS) [119,120]. The serum levels of miR-17, miR-34a, miR-155, and miR-373 have proven to be related to progression and metastasis of breast cancer [121]. Based on the existing evidence, two clinical trials to evaluate diagnostic potential of urinary miRNAs as well as blood miRNAs have been carried out [NCT03824613, NCT01391351]. miRNAs could be novel markers of cancer diagnosis and have enormous potential in clinical use in the future (Table 2).

## 6. Conclusions

miRNAs are key regulators of tumors. A plethora of studies revealed that plenty of miRNAs can regulate hallmarks of cancers including metastasis, therapy resistance, angiogenesis and tumor immunity. A therapeutic approach reversing abnormally expressed miRNAs is promising for cancer treatment and for the detection of abnormally expressed miRNAs might be used as diagnostic tools for cancer. Still, large cohort studies are required to validate the effect of miRNA-based diagnostic tools or therapeutic potential as well as miRNA-based drug safety. For the long haul, it is essential to find cancer specific miRNAs for better specificity as well as accuracy for cancer diagnosis as it is challenging to apply miRNAs-based therapy for cancer treatment. 

## Figures and Tables

**Table 2:** miRNAs with diagnostic potential in cancers.

Disease	miRNAs	Samples	Significances	Ref.
Barret’s esophagus	miR-192-5p, miR-194-5p and miR-215-5p	serum	The area under the curve (AUC) to diagnose BE by combining of these three miRNAs can reach 0.96–0.97.	[113]
Lung cancer	miR-146a-5p, miR-324-5p, miR-223-3p and miR-223-5p	bronchial epithelium	These four miRNAs are remarkably downregulated in lung cancer patients.	[114]
epithelial ovarian cancer	miR-200b	serum	High level of miR-200b can predict poor overall survival of patients.	[80]
Osteosarcoma	miR-221 and miR-222	serum	The upregulation of these two miRNAs is related with higher metastasis risk and poor prognosis.	[116]
Hepatocellular carcinoma (HCC)	miR-16	serum	The different expression of miR-16 can significantly increase the sensitivity of diagnosis.	[117]
Hepatocellular carcinoma (HCC)	miR-618 and miR-650	urine	The upregulation of these two miRNAs can increase the sensitivity of the diagnosis of HCC patients.	[118]
Breast cancer	miR-155	serum	miR-155 can act as a sensitive biomarker for monitoring the change in tumor size of breast cancer.	[119]
Breast cancer	miR-17, miR-34a, miR-155 and miR-373	serum	These four miRNAs showed significantly different expressions in serum of breast cancer patients.	[121]

## Data Availability

The data presented in this study are available upon request from the corresponding authors.
